# The association between SMAD7 polymorphisms and colorectal cancer susceptibility as well as clinicopathological features in the Iranian population 

**Published:** 2020

**Authors:** Zahra Akbari, Nahid Safari-Alighiarloo, Hamid Asadzadeh Aghdaei, Mohsen Vahedi, Mahdi Montazer Haghighi, Maryam Matani Borkheili, Ehsan Nazemalhosseini-Mojarad, Mohammad Reza Zali

**Affiliations:** 1 *Gastroenterology and Liver Diseases Research Center, Research Institute for Gastroenterology and Liver Diseases, Shahid Beheshti University of Medical Sciences, Tehran, Iran*; 2 *Endocrine Research Center, Institute of Endocrinology and Metabolism, Iran University of Medical Sciences, Tehran, Iran *; 3 *Basic and Molecular Epidemiology of Gastrointestinal Disorders Research Center, Research Institute for Gastroenterology and Liver Diseases, Shahid Beheshti University of Medical Sciences, Tehran, Iran*; 4 *Department of Epidemiology and Biostatistics, School of Public Health, Tehran University of Medical Sciences, Tehran, Iran*

**Keywords:** SMAD7, Single nucleotide polymorphism, Colorectal cancer

## Abstract

**Aim:**

Our aim was to investigate the association between two single nucleotide polymorphisms (SNPs) of SMAD7 and the risk of CRC among Iranian individuals.

**Background:**

Genome-wide association studies (GWAS) have identified 18q21 as a risk locus for colorectal cancer (CRC), which maps to the SMAD7 gene.

**Methods:**

This case–control study was conducted on 109 CRC cases and 109 controls in the Iranian population to evaluate the influence of two SNPs of SMAD7, rs2337106 and rs6507874, on the risk of CRC as well as on clinicopathological features. Genotype determination was performed by TaqMan assay via an ABI 7500 Real Time PCR System (Applied Biosystems) for the DNA of peripheral blood. Descriptive analysis and logistic regression model were used for statistical analyses.

**Results:**

Genotyping of the SNPs in the SMAD7 gene revealed that the frequency of G allele of rs2337106 was 53.7% in controls and 56.4% in cases (p-value=0.564) while the frequency of C allele of rs6507874 was 55.5% in controls and 56.3% in cases (p-value=0.772). Further, there were no significant differences in genotype frequencies of these SNPs between CRC patients and controls. The SMAD7 genotypes were not associated with the risk of CRC or with any clinicopathological characteristics such as tumor site, tumor grade, and stage TNM in CRC patients (p-value>0.05), even after adjustment for sex, age, and smoking status.

**Conclusion:**

Our results provided the first evidence that SMAD7 genotypes, rs2337106 and rs6507874, could not be predisposing markers in genetic susceptibility to CRC in an Iranian population, at least in the studied population.

## Introduction

 Colorectal cancer (CRC) is the third most common cancer and one of the major causes of cancer-related death worldwide (1). Over the last two decades, the incidence of CRC has notably increased in developed Asian countries such as Iran and could be now compared to that of Western countries (2-4). Accordingly, it has become regarded as a major worldwide health concern in our population. The complex etiology of this disease has not yet fully elucidated. Although environmental factors have a pivotal role on CRC incidence, early onset and familial clustering of CRC indicate a basic genetic contribution to colorectal carcinogenesis (5, 6).

 The SMAD7 (Mothers Against Decantaplegic Homolog 7) gene encodes an intracellular protein capable of binding to the transforming growth factor (TGF) - β type I receptor and preventing TGF-β-associated SMAD signaling (7). It has been proved that the TGF-β signaling pathway has significant roles in tumor initiation, invasion, and metastasis (8). This pathway plays a paradoxical role in cancer development and progression. Indeed, it acts as a tumor suppressor in the initial stages of tumorigenesis while promoting the invasion and metastasis of tumor cells in the later stages (9). SMAD7 antagonizes TGF-β signaling pathway through multiple mechanisms both in the cytoplasm and in the nucleus (10, 11). It inhibits TGF-β signaling by preventing the formation of the SMAD2/ SMAD4 complex (12). SMAD7 also plays other important roles in the etiology of CRC, such as interacting with b-catenin to regulate MYC expression and WNT signaling (13). Further, SMAD7 inhibits ERK1/2, JNK1/2, and p38 MAPKs under some circumstances related with tumorigenesis, such as erythroid differentiation and chondrocyte differentiation (14).

 Some studies have recognized SMAD7 gene (18q21) as a modest locus, but highly significant increase in CRC risk (15, 16). For instance, Broderick et al. conducted a genome-wide association study and identified three polymorphic variants in intron 3 of SMAD7 (rs4464148, rs4939827, and rs12953717) which were associated with CRC adenomas and carcinomas (15). In another study, Fortini et al. suggested that the associated CRC risk at 18q21.1 is due to four functional variants (rs6507874, rs6507875, rs8085824, and rs58920878) which regulate SMAD7 expression and potentially perturb a BMP negative feedback loop in TGFb/BMP signaling pathways (17). 

 In the present study, our aim was to explore the role of two SNPs of SMAD7, rs2337106 and rs6507874, in CRC risk assessment, assuming that there may be an association between those SNPs and CRC in the Iranian population. Clinical data were also used to study the correlation between them and mentioned SNPs in CRC development. Factors such as an age, sex, tumor site, tumor grade, and stage were evaluated. 

## Methods

**Study subjects and SNP genotyping**

The blood samples used in this study were collected from 109 CRC patients and 109 cancer-free subjects attending the Gastroenterology and Liver Disease Research Center, Shahid Beheshti University of Medical Science, Tehran, Iran. The inclusion criteria for patients included histologically confirmed CRC by positive colonoscopy and pathology results for colon or rectum malignant tumor, with no limitation on gender, age, or disease stage. Clinicopathological characteristics such as age, sex, tumor histology, and TNM stage were extracted from CRC patients’ medical records and histopathology reports. The histological classification and pathological staging were determined based on the criteria of the UICC Tumor-Node-Metastasis classification of Malignant Tumors (TNM) 6Th edition, 2002, colon and rectum (ICD-O C-18-C20). The controls were cancer-free individuals and randomly selected in the Iranian population. All control subjects showed no colonoscopy report for malignancy, inflammatory ulcers, or polyps, and they had no family history of gastrointestinal defects. A written informed consent was obtained from each participant at recruitment; demographic information including gender, age, and smoking was also gathered.

 Genomic DNA was extracted from peripheral blood using a phenol-chloroform standard protocol (25). Agarose gel-electrophoresis was used to assay the quality of genomic DNA, after which the concentration was quantitated by NanoDrop1000. Candidate SNPs, rs2337106 and rs6507874 were genotyped using TaqMan Real-Time Polymerase Chain Reaction (PCR) assay (C-152556-10 and C-29019586-10; Applied Biosystems; Foster City, CA). The reaction was performed on an ABI 7500 Real Time PCR System (Applied Biosystems). The PCR program was heated to 95°C for 10 minutes and 40 cycles of 92°C for 15 seconds and 60°C for 1 minute. Detection of fluorophores (released from the annealed probes due to the exonuclease activity of the polymerase during elongation) was performed at the end of the combined annealing-elongation step of 60°C. SDS software version 1.3 (Applied Biosystems) was utilized to identify individual genotypes.

**Statistical analysis**

Hardy–Weinberg equilibrium (HWE) for genotypes of each candidate SNP was separately determined in the case and control samples using a goodness-of-fit χ2-test. The differences in quantitative and qualitative demographic variables were assessed using a Student’s t-test or a χ2 test, respectively. Distribution of the allele and genotype frequencies was evaluated by χ2 test. Clinicopathological characteristics were also compared by a χ2 test. Logistic regression analysis was used to calculate odds ratio (OR) with 95% confidence intervals (CI) to evaluate the association between SMAD7 SNPs and the risk of CRC as well as clinicopathological characteristics after adjusting for confounding factors including gender, age, and smoking. All the statistical analyses were conducted by statistical package for social sciences (SPSS) software, where all p-values were two sided with the statistical significance criteria of P<0.05. 

## Results

**Subjects’ characteristics**

The basic characteristics of 109 CRC cases and 109 healthy subjects are listed in table 1. Clinicopathological features of CRC patients are also reported in Table 2. The prevalence of CRC in patients was noticeably more in colon rather than in rectum (73.4% and 26.6%, respectively) and most of them showed no sign of metastasis (n=95; 87.2%). Histological differentiation of tumor grades indicated that the majority of tumors were found to be well differentiated (n=50; 45.9%), such that patients were classified in three grades of poor (5.5%), moderate (28.4%), and well grade (45.9%). Further, at the time of diagnosis, five classes were assigned to the patients’ group from I to IV with respect to tumor node metastasis (TNM) as 1.8%, 13.8%, 70.6%, and 10.1% respectively. 

**Table 1:** Characteristics of colorectal cancer patients and control groups

Characteristics	Patients (n=109)			Controls (n=109)			p^a^
	n	%	Mean (SD)	n	%	Mean (SD)	
Age (years)			60.31±11.76			44.32±16.28	<0.001
Gender							0.015
Male	62	56.9		44	40.4		
Female	47	43.1		65	59.6		
Smoking status							0.251
Never	90	82.6		96	88.1		
Current	19	17.4		13	11.9		

p^a^> 0.05 for differences between patients and controls

**Table 2:** Characteristics of colorectal cancer patients

Variables	N	%
Tumor grade		
Well	50	45.9
Moderate	31	28.4
Poor	6	5.5
Not determined	22	20.2
Location		
Colon	80	73.4
Rectum	29	26.6
TNM Stage		
I+II	64	58.7
III+IV	45	41.3
T		
T1	2	1.8
T2	15	13.8
T3	77	70.6
T4	11	10.1
Unknown	4	3.7
N		
N0	61	56.0
N1	26	24.0
N2	11	10.0
Unknown	11	10.0
M		
M0	95	87.2
M1	14	12.8
Dukes stage		
A	1	0.9
B	60	55.0
C	33	30.3
D	15	13.8

**Genotypic and allelic frequencies**

**Figure 1:**
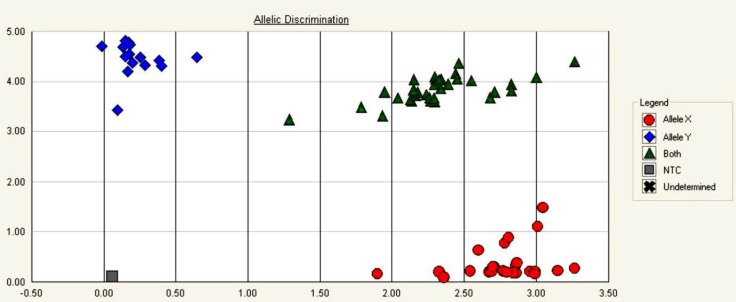
Allelic discrimination plot representing rs6507874 genotypes with four symbols (squares, Diamonds, triangles, and circles) for NTC sample, CC, CT, and TT genotypes respectively; The x-axis is the extent of emission from FAM channel and the y-axis represents emission from VIC channel

**Figure 2:**
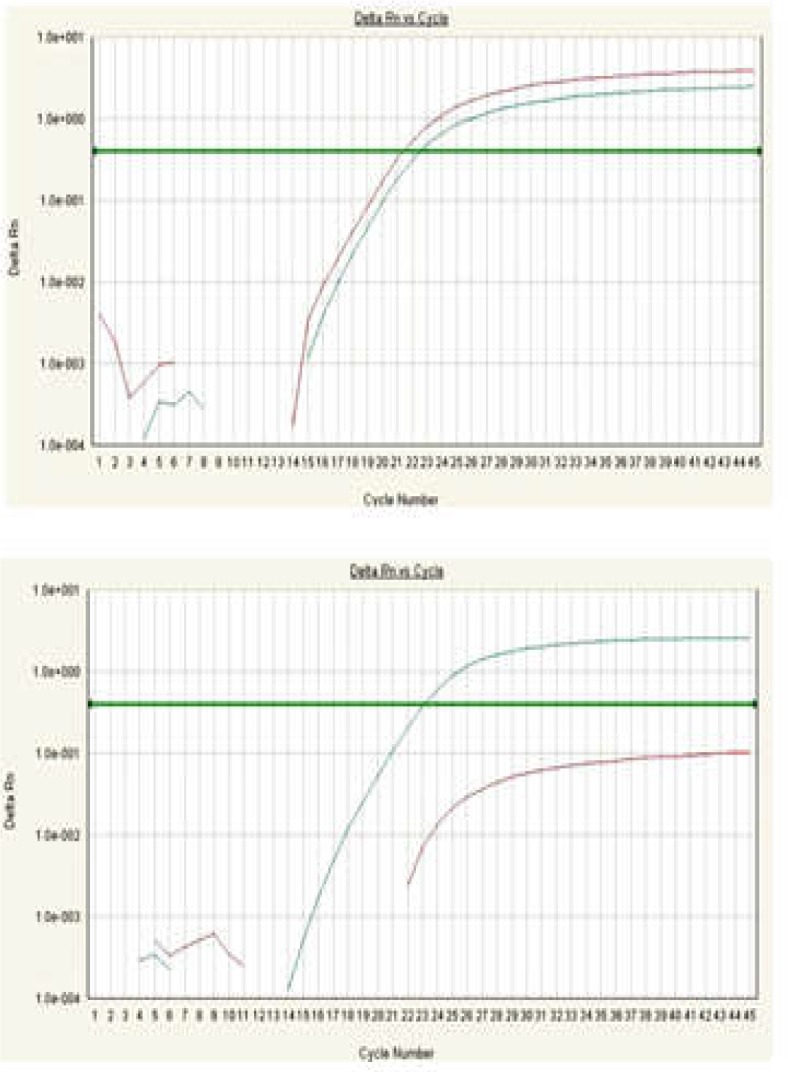
Amplification plots of rs6507874 produced by the SDS analysis software; The x-axis is the amplification cycle number while the y-axis represents raw fluorescent value. A) Example of a true heterozygote (CT) with the amplification curve for both fluorophore channels (VIC, FAM). B) Example of a true homozygote (CC) with the amplification curve of the VIC channel

 The allele frequency and genotype distribution of these SNPs of SMAD7 are summarized in Table 3. Also, some representative allelic discrimination plots are presented in Figures 1 and 2. Genotype frequencies of two SNPs for each group separately were in accordance with Hardy–Weinberg equilibrium (all p > 0.05, Table 3). These indicated no population stratification and sampling bias. Genotyping of the SNPs in the SMAD7 gene revealed that the G allele of rs2337106 was found in 53.7% of controls and 56.4% of cases; the C allele of rs6507874 was observed in 55.5% of controls and 56.3% of cases. There were no significant differences in genotypes and allelic distributions of these genetic polymorphisms of SMAD7 between CRC patients and control groups. Further, these two SNPs in SMAD7 were not significantly associated with CRC risk in Iranian samples (Table 3). For the rs2337106, the OR of the additive model was 1.340 (0.619-2.929), and that of the dominant model was 1.505 (0.766-2.954). On the other hand, the OR of the additive model for the rs6507874 was 1.026 (0.455-2.312) and that of the dominant model was 0.822 (0.405-1.671). 

**Table 3:** The genotype and allele frequencies of SMAD7 gene rs2337106 and rs6507874 polymorphisms among CRC patients and control

SNPs ID Genotypes	Patients no.(%)^a^ (n=109)	Controls no.(%)^a^ (n=109)	P-value^b^	OR (95% CI)	
				Crude Adj.^b^	
rs 2337106					
CC	18 (16.5 )	25 (22.9)	0.541	1.00 (Reference)	1.00 (Reference)
CGc	59 (54.1)	51 (46.8)	0.300	1.607 (0.788-3.276)	1.538 (0.681-3.474)
GGc	32 (29.4)	33 (30.3)	0.331	1.340 (0.619-2.929)	1.562 (0.635-3.842)
CG or GGd	91 (83.5)	84 (77.1)	0.268	1.505 (0.766-2.954)	1.547 (0.715-3.347)
C allele	95 (43.6)	101 (46.3)	0.564	1.00 (Reference)	-
G allele	123 (56.4)	117 (53.7)		1.118 (0.766-1.630)	-
rs 6507874					
TT	20 (18.3)	17 (15.6)	0.343	1.00 (Reference)	1.00 (Reference)
TCc	54 (49.5)	63 (57.8)	0.208	0.729 (0.347-1.530)	0.570 (0.238-1.368)
CCc	35 (32.1)	29 (26.6)	0.724	1.026 (0.455-2.312)	0.843 (0.328-2.168)
TC or CCd	89 (81.7)	92 (84.4)	0.327	0.822 (0.405-1.671)	0.659(0.286-1.517)
T allele	94 (43.1)	97 (44.5)	0.772	1.00 (Reference)	-
C allele	124 (56.9)	121 (55.5)		1.057 (0.724 -1.544)	-

The observed genotype distribution of patients and controls were in agreement with the Hardy-Weinberg equilibrium;b Adjusted for age, sex and smoking status; c Additive genetic model ; d Dominant genetic model

**Table 4:** Association between genotypes of the SMAD7 SNPs and tumor location plus grade

Characteristics	rs2337106			P-value	rs6507874			P-value
	CC	CG	GG		TT	TC	CC	
Tumor Location				0.407				0.508
Colon	11 (13.7)	44 (55.0)	25(31.3)		16(20.0)	37 (46.2)	27 (33.8)	
Rectum	7 (24.1)	15 (51.7)	7 (24.1)		4 (13.8)	17 (58.6)	8 (27.6)	
Tumor grade				0.813				0.743
Well	8 (16.0)	26 (52.0)	16 (32.0)		8 (16.0)	29 (58.0)	13 (26.0)	
Moderate	7 (22.6)	15 (48.4)	9 (29.0)		6 (19.3)	13 (42.0)	12 (38.7)	
Poor	1 (16.7)	3 (50.0)	2 (33.3)		2 (33.3)	2 (33.3)	2 (33.3)	
Not determined	2 (9.1)	15 (68.2)	5 (22.7)		4 (18.2)	10 (45.4)	8 (36.4)	

Values are expressed as number ((%; P-value is adjusted for age, sex and smoking status

**Table 5:** Association of genotypes of the SMAD7 SNPs with Dukes and TNM stage

Characteristics	rs2337106			P-Value	rs6507874			P-Value
	CC	CG	GG		TT	TC	CC	
TNM Stage				0.397				0.644
I+II	13 (20.3)	32 (50.0)	19 (29.7)		10 (15.6)	32 (50.0)	22 (34.4)	
III+IV	5 (11.1)	27 (60.0)	13 (28.9)		10 (22.2)	22 (48.9)	13 (28.9)	
T				0.504				0.073
T1	1 (50.0)	0 (00.0)	1 (50.0)		0 (0.0)	0 (0.0)	2 (100.0)	
T2	4 (26.7)	5 (33.3)	6 (40.0)		2 (13.3)	8 (53.3)	5 (33.3)	
T3	12 (15.6)	44 (57.1)	21 (27.3)		15 (19.5)	41 (53.2)	21 (27.3)	
T4	1 (9.1)	7 (63.6)	3 (27.3)		3 (27.3)	5 (45.4)	3 (27.3)	
Unknown	0 (0.0)	3 (75.0)	1 (25.0)		0 (0.0)	0 (0.0)	4 (100.0)	
N				0.648				0.085
N0	12 (19.7)	32 (52.4)	17 (27.9)		10 (16.4)	31 (50.8)	20 (32.8)	
N1	2 (7.7)	14 (53.8)	10 (38.5)		5 (19.2)	13 (50.0)	8 (30.8)	
N2	3 (27.3)	6 (54.5)	2 (18.2)		4 (36.4)	7 (63.6)	0 (0.0)	
Unknown	1 (9.1)	7 (63.6)	3 (27.3)		1 (9.1)	3 (27.3)	7 (63.6)	
M				0.386				0.095
M0	17 (17.9)	52 (54.7)	26 (27.4)		18 (18.9)	50 (52.7)	27 (28.4)	
M1	1 (7.1)	7 (50.0)	6 (42.9)		2 (14.3)	4 (28.5)	8 (57.1)	
Dukes Stage				0.337				0.574
A	1 (100.0)	0 (0.0)	0 (0.0)		0 (0.0)	0 (0.0)	1 (100.0)	
B	11 (18.3)	32 (53.3)	17 (28.3)		10 (16.7)	29 (48.3)	21 (35.0)	
C	5 (15.1)	19 (57.6)	9 (27.3)		7 (21.2)	19 (57.6)	7 (21.2)	
D	1 (6.7)	8 (53.3)	6 (40.0)		3 (20.0)	6 (40.0)	6 (40.0)	

TNM, Tumor Node Metastasis; Values are expressed as number (%); P-value is adjusted for age, sex and smoking status

We also investigated the relationship between SMAD7 gene polymorphisms and the various clinicopathological features in CRC patients, with the obtained results shown in Tables 4 and 5. The genotype analysis of these SNPs of SMAD7 gene revealed that the CG genotype of rs2337106 (44± 55.0), (15±51.7) and TC genotype of rs6507874 (37± 46.2), (17±58.6) were the most prevalent genotypes in both colon and rectum cancer, respectively. Likewise, concerning the tumor grade, both genotypes (CG rs2337106) and (TC rs6507874) were more prevalent as well grade (26±52.0), (29±58.0) and moderate (15±48.4), (13±42.0), respectively. There were no significant associations between the genotype distribution of rs2337106 and rs6507874 polymorphisms and any of the clinical or pathological features (tumor localization, differentiation, and metastasis) (Tables 4 and 5). Hence, these polymorphic sites could not be candidates for predicting the clinicopathological features of CRC in our population.

## Discussion

SMAD7 is one of the important effector of TGF-β signaling whose impaired expression has been documented to influence CRC progression (18, 19). Here, we studied the association between two genetic polymorphisms of SMAD7, rs2337106 and rs6507874, and the risk of CRC in an Iranian population. Also, the associations between these polymorphisms and clinicopathological factors such as tumor location, TNM stage, and tumor grade was also investigated. The results showed no significant differences in genotype frequencies of these SNPs between CRC patients and controls. There were no associations either between these polymorphisms and the risk of CRC or clinicopathological factors in our population. 

It has been suggested that SMAD7 can switch from tumor-suppressive to tumor-promoting depending on the tumor stage. Since TGF-β signaling pathway plays contradictory roles in early versus advanced tumor stages, the switch-in role of SMAD7 is not surprising. Further, the SMAD7 has interaction with a vast array of functionally heterogeneous molecules that may be differently expressed during the carcinogenetic process (7). This role alteration may have an association with the tumor microenvironment and/or somatic mutation (20). Various studies have suggested different expression of SMAD7 in human cancers, which could either sustain or restrain cancer cell growth  (21, 22). There are several studies demonstrating the SMAD7 as a risk factor in CRC. Stolfi et al. reported that SMAD7 has a noticeable role in sustaining colon tumorigenesis (23). From early on, the study results of Boulay et al. revealed that the deletion of SMAD7 in CRC patients had a promising clinical outcome compared with patients with SMAD7 amplification (24). Also, Halder et al. found that SMAD7-overexpressing FET cells showed aggressive colony formation on soft agar and increased tumorigenicity *in vivo* in terms of controlling FET cells (25). Conversely, the opposing role of SMAD7 in the control of sporadic and colitis-associated CRC has been shown by one study; they reported that over-expression of SMAD7 in T cells is associated with severe colitis and reduces the growth of colitis-associated CRC (26).

 Although the number of polymorphisms of SMAD7 gene have been associated with increased risk of CRC development (14, 15, 27, 28), our selected SNPs, rs2337106 and rs6507874, did not have any significant associations with CRC susceptibility. The results of some studies were in line with our findings and some were not. L.Slattery and colleagues in 2010 reported that there was no association between rs2337106 of SMAD7 gene and colon cancer in Western area of the United States (28). After a while in 2013, the results of Jiang. X et al. studies showed no significant relationship either between this SNP and CRC in some areas of the United States (29). Regarding the involvement of another SNP, rs6507874, Alemn et al. inspected all polymorphisms within the 17-kb region of the 18q21 locus; based on linkage disequilibrium (LD), it protects the disease-causing variants responsible for the SMAD7–18q21 association with CRC. Their results showed that rs6507874, not alone but together with 24 other SNPs, had an association with the development of CRC at the 5% statistical threshold (30). In another study, rs6507874 was introduced as one of functional variants that regulates SMAD7 expression and is implicated in the risk of CRC.

 In the present study, we found that C allele in rs6507874 is the most frequent allele in our population, which is similar to other populations such CEU in Western United States (53%) and Yoruba in Ibadan, Nigeria (59%); as also, G allele in rs2337106 was a more frequent allele in our study which is in line with other populations such as Japanese in Tokyo, Japan (50.4%) and Iberian Population in Spain (55.1%). These comparisons have been made based on the results of 1000 Genomes Project, (https://www.ncbi.nlm.nih.gov/variation/tools/1000genomes/). Geographic or ethnic variations and environmental factors should be the possible reasons for such discrepancies in allele frequencies across different populations. 

 Note that for the first time, the association between SMAD7 genotypes and clinicopathological characteristics was computed in our population. Our results revealed no significant association between rs2337106 or 6507874 genotypes and clinicopathological in CRC patients. Mates et al. in 2012 investigated the association between several SNPs and tumor site as well as staging features in CRC. They found that carriers of risk alleles at loci rs4939827 of SMAD7 gene could harbor increased susceptibility to development of rectal cancer rather than colon cancer (31). According to our results, however, we cannot recommend that these polymorphisms of SMAD7 gene would be associated with progression or metastasis of CRC in an Iranian population.

 The strength of this study was using well-defined homogenous samples with detailed clinical data, though a relatively small sample size was one of our limitations. In addition, two polymorphisms of SMAD7 gene were studied in our population, which is not sufficient to cover the entire gene. Given that gene-gene interactions and interactions between different loci on the same gene may affect the risk of complex diseases, thus our data are preliminary in CRC studies.

Conclusion: The results of this study indicated no evidence of association between two polymorphisms of SMAD7 gene, rs2337106 C/G and rs6507874 T/C, and the risk of initiation or development of CRC. No significant effect was found either for these SNPs on clinicopathological features in patients with CRC. Accordingly, these polymorphisms could not be major risk factors for CRC in our population. Notably, this is the first case-control study to have examined the influence of two polymorphisms of SMAD7 gene on clinicopathological features and CRC risk in an Iranian population. Since GWAs studies have suggested significant associations between SMAD7 and the risk of CRC, other SMAD7 SNPs should be further investigated in future studies. In addition, the results were found with some unavoidable limitations such as small sample size. Thus, larger study groups and various populations should be the effective options to clarify the exact relationship between these SNPs and CRC development.
